# Fatty acid metabolism of immune cells: a new target of tumour immunotherapy

**DOI:** 10.1038/s41420-024-01807-9

**Published:** 2024-01-20

**Authors:** Sheng Zhang, Kebing Lv, Zhen Liu, Ran Zhao, Fei Li

**Affiliations:** 1 Center of Hematology, The First Affiliated Hospital of Nanchang University, Nanchang, China; 2 Jiangxi Clinical Research Center for Hematologic Disease, Nanchang, China; 3 Institute of Lymphoma and Myeloma, Nanchang University, Nanchang, China

**Keywords:** Immunotherapy, Cancer microenvironment, Cell biology

## Abstract

Metabolic competition between tumour cells and immune cells for limited nutrients is an important feature of the tumour microenvironment (TME) and is closely related to the outcome of tumour immune escape. A large number of studies have proven that tumour cells need metabolic reprogramming to cope with acidification and hypoxia in the TME while increasing energy uptake to support their survival. Among them, synthesis, oxidation and uptake of fatty acids (FAs) in the TME are important manifestations of lipid metabolic adaptation. Although different immune cell subsets often show different metabolic characteristics, various immune cell functions are closely related to fatty acids, including providing energy, providing synthetic materials and transmitting signals. In the face of the current situation of poor therapeutic effects of tumour immunotherapy, combined application of targeted immune cell fatty acid metabolism seems to have good therapeutic potential, which is blocked at immune checkpoints. Combined application of adoptive cell therapy and cancer vaccines is reflected. Therefore, it is of great interest to explore the role of fatty acid metabolism in immune cells to discover new strategies for tumour immunotherapy and improve anti-tumour immunity.

## Facts

1. The reprogramming of fatty acid metabolism of immune cells can directly affect the activation and differentiation of immune cells to affect the host’s anti-tumor immunity;
2. The disorder of lipid metabolism in cancer is an important factor affecting tumor progression and changing TME intercellular communication.
3. Different types of fatty acid metabolism in different immune cell subsets will affect the function of immune cells.
4. The study on the relationship between fatty acid metabolism and tumor immunotherapy has shown significant efficacy in many types of tumor therapy.

## OPEN QUESTIONS

1. How does disruption of fatty acid metabolism affect cell communication in the tumor microenvironment?
2. Is the fatty acid metabolism of targeted immune cells targeted at a single stage?
3. Does fatty acid metabolism have good therapeutic potential in tumor immunotherapy?

## Introduction

After decades of research on cancer, the factors that affect cancer progression are referred to as characteristics of cancer [1]. In the background of the widespread occurrence of tumours, the serious effects of metabolic changes and immunosuppression of tumour cells have been broadly studied. Indeed, researchers consider the metabolic reprogramming of immune cells, which can directly affect activation and differentiation of immune cells or indirectly change the function of immune cells by affecting transcription, as a new marker for cancer [2]. A growing body of research has provided ample evidence that tumour immune escape is associated with metabolic reprogramming of the TME, in which cancer cells need to respond to hypoxia, acidification, and limited nutritional support while supporting their growth and metastasis [3]. Lipid metabolism, especially fatty acid metabolism, has been gradually recognized as a crucial part of metabolic processes, playing an important role in the survival and proliferation of tumour cells and supporting the differentiation and migration of immune cells in the TME [4]. Therefore, the TME can reprogramme the fatty acid metabolism of immune cells to influence the host’s anti-tumour immunity [5]. The changes in lipid metabolism in tumour cells and immune cells in the TME reshape the lipid composition and further link the related metabolic characteristics of tumours and other cells [6]. As an important component of triglycerides (TGs), phospholipids and glycolipids, fatty acids provide energy or cellular signal support for almost all cells to maintain normal (or abnormal) physiological and pathological functions. Therefore, the combination of metabolic targets and immunotherapy has great potential and prospects.

The immune cells in tumours are regulated by the cancer cells to protect them from being killed by the immune system, a situation that has aroused great interest, and the resulting immunotherapy has shown an unusual effect in the fight against cancer. At present, immunotherapy mainly includes immune checkpoint blockade (ICB), adoptive cell therapy (ACT), and tumour vaccines, among others [7]. Despite great progress as a therapeutic strategy to promote the immune response to tumour antigens, its clinical application is limited because it is only effective for a small number of patients due to immunosuppression, tumour heterogeneity, drug resistance and other problems. In this review, we mainly discuss the fatty acid metabolism of immune cells in the TME and explore the goal of providing more therapeutic strategies.

## Fatty acid metabolism in the tumour microenvironment

The survival, proliferation and metastasis of cancer cells in hypoxia, acidification and other harsh environments depend on metabolic reprogramming. Various changes in cellular metabolism contribute to tumour progression, invasion, and metastasis and even regulate the resistance of cancer cells to treatment [8, 9]. However, tumour cells can adapt to changes in the environment and use different metabolic patterns to ensure their growth in harsh environments to cope with oxidative stress and drug exposure. Researchers regard this interesting metabolic adaptation as a self-protection measure for cancer cells against treatment before mutation and regeneration. Overall, targeting the metabolism of tumour cells should be an effective way to improve anticancer treatment or to achieve re-sensitization cancer to drugs, though it remains unclear whether the metabolic changes are related to drug resistance.

The Warburg effect, whereby cancer cells show stronger glucose uptake than normal tissues, was proposed as early as the 1920s; it was further found that through glycolysis, cancer cells are more likely to convert glucose to lactic acid, regardless of oxygen content [10]. As the earliest discovered adaptive metabolism, glucose metabolism through the glycolysis pathway provides a carbon source for energy and key biochemical precursor synthesis in cancer, and some tumours obtain carbon and amino nitrogen by increasing glutamine consumption [11]. Thus far, it is generally agreed that the ability of tumour lipid synthesis is enhanced and that it is closely related to glucose metabolism. The disorder of lipid metabolism in cancer is an important factor affecting cancer progression and changing intercellular communication in the TME [12–16]. Indeed, the increase in exogenous FA synthesis and uptake and synthesis of endogenous FA in highly proliferative cancer cells and the TME are important manifestations of lipid metabolic adaptation [17–19]. Fatty acids and other lipids (triglycerides, phospholipids, sterols, etc.) in cancer cells provide energy, membrane components and signal molecular composition and are involved in the regulation of energy supply, proliferation, metastasis, environmental adaptation and therapeutic responses [17, 20, 21].

Fatty acid metabolism includes de novo synthesis of fatty acids, fatty acid decomposition (mainly β-oxidation) and the transport of metabolites. Under the background of cancer biology, a large number of studies have shown that de novo synthesis of fatty acids plays a critical role in activation of carcinogenesis and is the main way for cancer cells to obtain fatty acids [22–24]. However, recent research suggests that some tumour cells and tissues are simultaneously able to utilize lipid synthesis and breakdown (including lipolysis and lipophagy) pathways to obtain fatty acids, further supporting their survival and proliferation [25–27].

**Fig. 1:**
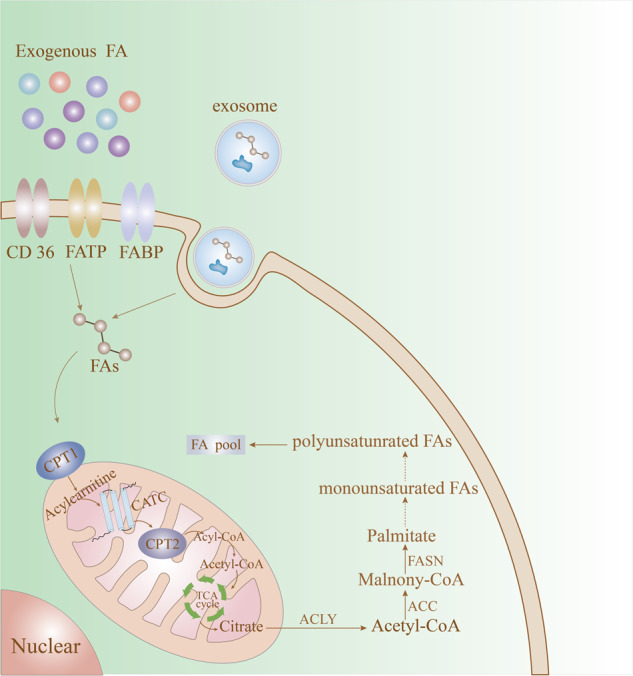
Fatty acid metabolism of tumour cells. Tumour cells absorb fatty acids by expressing FAT (also known as CD36), FATPs and FABP on the plasma membrane and exosome transport and carry out β-oxidation and the tricarboxylic acid cycle.

Glycolysis, the tricarboxylic acid (TCA) cycle, and the pentose phosphate pathway (PPP) provide the major substrate for intracytoplasmic fatty acid synthesis, namely, (FAS)-acetyl-CoA [28]. Acetyl-CoA carboxylases (ACCs) are rate-limiting enzymes in de novo synthesis of fatty acids, regulating the first step of conversion of acetyl-CoA carboxylate to malonyl-CoA at a 7:1 ratio, which is catalysed by fatty acid synthase (FASN), to produce saturated palmitate (FA16:0) [17]. Saturated palmitate is then elongated or desaturated to other saturated or unsaturated long-chain fatty acids (LCFAs). In addition, fatty acids can be condensed with glycolysis-derived glycerols to produce various combinations of triacylglycerols and phospholipids as key components of many cellular structures [29]. β-Oxidation in mitochondria is the main oxidation mode of fatty acid decomposition, providing much energy. Among them, short-chain fatty acids (SCFAs) and medium-chain fatty acids (MCFAs) can directly enter mitochondria, whereas long-chain fatty acids (LCFAs) depend on the carnitine shuttle system and are first converted to fatty acyl-CoA with the assistance of fatty acyl-CoA synthetase. Fatty acyl coenzyme A is converted to fatty acyl carnitine under the catalysis of the rate-limiting enzyme carnitine palmitoyltransferase 1 (CPT1) [30] and enters mitochondria with the help of carnitine/acylcarnitine translocase (CACT). The process from acyl carnitine to acyl coenzyme A is completed under the catalysis of carnitine palmitoyltransferase 2 (CPT2), followed by decomposition to acetyl coenzyme A in mitochondria via multiple steps. Acetyl-CoA then enters the tricarboxylic acid cycle (TCA) to produce ATP [31]. Cellular fatty acid uptake depends on specific transporters expressed on the plasma membrane, including FA transporter (FAT), the FA transporter family (FATPs) and plasma membrane FBP-binding protein (FABP) [32] (Fig. 1). Recent studies have also found that tumour cells can absorb fatty acids through exosome transport, which is not described in this review [33].

## Fatty acid metabolism of immune cells

There is evidence that immune system function is related to fatty acids, energy supply and nutritional support at the cellular level [34, 35]. The combination of nutritional availability and different metabolic states generally determines the executable energy-consuming activities of different immune cells. Metabolic plasticity may be the key factor for the immune system to adapt to changes in the microenvironment [36]. Researchers believe that immune cells have different metabolic characteristics; specifically, different immune cell subsets possess different metabolic characteristics [37–39]. Metabolic processes, including glycolysis, the citric acid (TCA) cycle, the pentose phosphate pathway (PPP), fatty acid synthesis and oxidation, amino acid metabolism and oxidative phosphorylation, may be changed to meet the energy requirements of different immune cell subsets for functional growth and proliferation [40]. This means that fatty acids affect immune cell function, regardless of their type; extracellular fatty acids can then be recognized and absorbed into the cells through the above-mentioned process, becoming the substrate for β-oxidation and complex lipid synthesis as well as nuclear receptor signals [41, 42]. In this review, we focus on the role of fatty acid metabolism in different immune cell subsets and put forward new insights into the possible role of fatty acids in immune metabolism (Table 1).

**Table 1:** Fatty acid metabolism of immune cells in the TME.

Model	Immune cells	Changes of fatty acid metabolism	Effect	Reference
Breast cancer	CD8^+^ Teff cells	Increase FAO	Inhibition of CD8^+^ T-cell effects and tumour growth	[47]
Melanoma and pancreatic cancer	CD8^+^ CTLs	Add short-chain fatty acids (pentanoate and butyrate)	Elevate production of effector molecules, enhance anti-tumour activity	[55]
Prostate cancer	eTregs	Decrease expression of genes related to fatty acid synthesis and the content of free fatty acids in eTreg cells.	Promote TIL anti-tumour responses	[74]
Melanoma	Tregs	Increase FA uptake	Reduce the metabolic pressure caused by the TME	[82]
Prostate cancer	Macrophages	PGE3 selectively promotes M2a polarization	Anti-inflammatory and anti-tumour effects	[97]
Breast, colon and prostate cancer	Macrophages	Increase FA uptake and up-regulate FAO	Promote differentiation to M2-like TAMs, anti-tumour effects	[91]
Hepatocellular cancer	DC cells	Down-regulated expression of enzymes involved in FAS and inhibited fatty acid synthesis	Immunosuppression	[126]
Melanoma	DC cells	Increase FAO	Promote immune evasion	[155]
Lung and colon cancer	MDSC	Increase FA uptake and up-regulate FAO	Enhance immunosuppressive function	[147]

*TME* tumour microenvironment, *Teff cell* effector T cell, *FAS* fatty acid synthesis, *FA* fatty acid, *FAO* fatty acid oxidation, *Treg cell* regulatory T cell, *DC cell* dendritic cell, *TAM* tumour-associated macrophage, *MDSC* myeloid-derived suppressor cell, *TIL* tumour-infiltrating lymphocyte.

### CD8+ T cells and fatty acid metabolism

T cells with immune activity are important factors involved in anti-tumour immunity. Fatty acid metabolism is of great significance to T cells. De novo synthesis of fatty acids provides raw materials for their proliferation, and fatty acid oxidation provides energy for them and plays an important role in the differentiation of memory function of CD8+ T cells and regulation of subset differentiation of CD4+ T cells [43]. As the most important lymphocyte subset in the TME, CD8+ T cells, also known as CTLs, mainly destroy cancer cells by releasing granzymes, perforins and other effectors. Maintenance of their effector function generally requires the assistance of aerobic glycolysis. However, due to the hypoxia or low glucose microenvironment in cancer, CD8+ T cells increase fatty acid uptake and catabolism and initiate fatty acid oxidation [44].

Researchers have focused on regulation of fatty acid anabolism in T cells [45, 46], and interestingly, different studies have reported opposite results. Lipid metabolism in CD8+ T cells may play an immunosuppressive role. In breast cancer, leptin/STAT3 signalling is driven by obesity, which aggravates fatty acid oxidation (FAO) and reduces glycolysis in CD8+ T cells through the PD-L1 channel, which leads to inhibition of effector function and tumour growth [47]. In another study, PD-L1-mediated blockade of T-cell differentiation into effector subsets was mediated by inhibiting glycolysis and promoting fatty acid oxidation [48]. Earlier experiments have suggested that the activity and function of CD8+ T cells decreases significantly after treatment with tumour-derived free fatty acids [49, 50]. However, in gastric cancer, the lack of fatty acids may cause the memory T cells residing in tissues to lose their functions because they mainly rely on fatty acid oxidation for energy supply [51]. IL15 can up-regulate expression of carnitine palmitoyltransferase-1A (CPT1A), the key rate-limiting enzyme of fatty acid oxidation, and increase the spare respiration capacity (SRC) of CD8+ T memory cells to cope with additional stress [52]. In another study, expression of CPT1A was up-regulated through the CD28 costimulatory signalling pathway, which can enhance fatty acid oxidation and mitochondrial respiratory function [53].

By examining the immunophenotype of the TME in a mouse breast cancer model, it was shown that the new selective inhibitor of phosphatidylinositol 3-kinase α (PI3Kα) CYH33 can weaken the inhibition of CD8+ T cells mediated by M2-like macrophages by reprogramming macrophages and promote the metabolism of FA in tumour tissue to enhance infiltration and activity of CD8+ T cells, exerting an anti-tumour effect [54]. The regulation of immune activity by short-chain fatty acids (SCFAs) may be achieved by inducing metabolic changes. Studies have revealed that valerate and butyrate significantly enhance the anti-tumour activity of cytotoxic T lymphocytes (CTLs) and chimeric antigen receptor (CAR) T cells by increasing the production of effector cytokines (CD25, IFN-γ and TNF-α) in CD8+ T cells. At the same time, the function of mTOR as a metabolic sensor of central cells was increased [55]. Due to the shift of mammalian rapamycin complex target 1 (mTORC1) and mTORC2 signal inhibition of the immune response from effector to memory [56, 57], the importance of T-cell activation and enhancement of mTOR signalling may also be caused by positive feedback through fatty acid metabolism, suggesting that targeted fatty acid metabolism may play a role in adoptive T-cell therapy.

Fatty acid catabolism supports the survival of effector CD8+ T cells under harsh TME conditions. Oxisome proliferation-activated receptor α (PPAR- α) is responsible for regulation of fatty acid oxidation and can restore part of the effector function of CD8+ TILs in low-glucose and hypoxic TMEs [44]. Prostaglandins are a group of unsaturated fatty acids produced by arachidonic acid enzymes. Studies have shown that metastatic mouse renal carcinoma (RECA) cells produce excessive prostaglandin eosin 2 (PGE_2), which inhibits initiation of tumour-specific CTLs; this is achieved by blocking the IFNγ signal between intercellular cell adhesion molecule-1 (ICAM-1) and lymphocyte receptor lymphocyte function-associated antigen (LFA-1) [58]. Bezafibrate is a complex agonist of proliferation-activated receptor γ (PPAR γ) co-activator 1-α (PGC-1α). By promoting expression of CPT1A, long-chain acyl-CoA dehydrogenase (LCAD) and fatty acid oxidation-related genes in CD8+ TILs, it maintains the number of CTLs and inhibits tumour progression in a lung cancer transplanted tumour model [59], which is consistent with the research of Saibil et al. [60]. However, due to simultaneous upregulation of glycolysis, further exploration is needed to attribute the results to fatty acid oxidation.

Researchers have long found that depletion of CD8+ T cells in the TME is one of the important factors restricting the effectiveness of tumour treatment. In depleted CD8+ T cells, inhibitory receptors such as cytotoxic T lymphocyte antigen-4 (CTLA-4), programmed death-1 (PD-1), T-cell immunoglobulin-3 (TIM-3) and lymphocyte activation gene 3 (LAG-3) are significantly up-regulated and affect the FA metabolism of T cells in the TME [61, 62]. PD-1 promotes fatty acid oxidation of T cells and increases expression of CPT1A [48]. In a study of non-small cell lung cancer, PD-1hiCD8+ TILs had a higher lipid content and uptake capacity than PD-1lowCD8+ T cells [63].

**Fig. 2:**
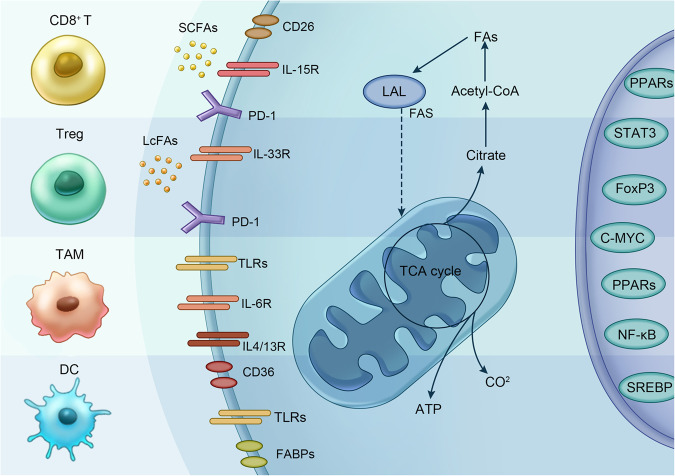
Targets of Fatty Acid Metabolism in immune cells. Immune cells (CD8^+^T cell, Treg, TAM, DC) in tumor microenvironment regulate the process of fatty acid metabolism through corresponding targets, which in turn affect the function of immune cells and their sensitivity to immunotherapy.

Moreover, all kinds of lipids present in the TME have been proven to have certain immunomodulatory effects. For example, CD36, an FA transport receptor and lipid oxidation scavenging receptor, increases expression of CD36 in melanoma and colon cancer cell lines, induces lipid peroxidation and increased fatty acid uptake, and causes functional inhibition and iron death in CD8+ CTL cells in a CD36-dependent manner (Fig. 2) [64, 65]. We will not talk too much about lipid peroxidation here, but it may be our next research direction. Fatty acid metabolism changes have a variety of effects on CD8+ T cells, and the above studies have shown that fatty acid metabolism plays an important role in the activation, differentiation and effector function of CD8+ T cells. These possible promotive or inhibitory effects depend on the type, source, and transport mode of fatty acids and interaction with CD8+ T cells. Therefore, more evidence in T-cell ab initio fatty acid synthesis, catabolism and uptake and other related experiments are needed with regard to the regulatory targets of T-cell function.

### Treg cells and fatty acid metabolism

Th1, Th2, Th17 and Treg cells are all subgroups that differentiate from immature CD4+ T cells under antigen stimulation and have different metabolic characteristics. After T cells first interact with antigen-presenting cells, TCR recognizes the ligands presented by MHC and activates with the help of the costimulatory signal CD28, relying on aerobic glycolysis to provide rapid production of ATP. At the same time, nucleotide production, lipid synthesis and a certain amount of Warburg effect are also necessary [66]. Mammalian target of rapamycin (MTOR) is the main target of the above metabolic mechanism, and phosphatidylinositol 3-kinase-protein kinase B (PI3K-AKT) signalling and c-Myc are the main pathways that induce its downstream regulation [67].

FAs have been shown to be involved in activating differentiation of CD4+ T-cell subsets [45, 68]. As a widely studied cancer-promoting factor, Twist-1 has the characteristics of increased FA oxidation and decreased glycolysis in heavily stimulated Th1 subsets. The purpose may be to protect lymphocytes from oxidative stress by promoting fatty acid oxidation [69]. Soraphen A, for example, can specifically inhibit acetyl-CoA carboxylase 1 (ACC1) to block Th17 differentiation, possibly by inhibiting de novo fatty acid synthesis of T cells [68], and differentiation and activation of naive CD4+ T cells into Th17 subsets depend on FA synthesis [70]. However, in the two groups of experiments, the Treg subgroup had different characteristics, suggesting that the Th17/Treg subgroup may carry out the opposite metabolic programme, and this subgroup seems to be able to regulate the metabolic target.

Such a change in fatty acid metabolism may also be an important factor affecting the uniqueness of Treg cells in the TME. Treg cells increase their glycolysis rate and lipid biosynthesis, and fatty acid oxidation helps to amplify their metabolic signals [71, 72]. Abnormally increased or decreased fatty acid synthesis and storage seem to be harmful to Treg cells, such as abnormal changes in triglycerides and inhibition or loss of lysosomal acid lipase. Increased storage of triglycerides leads to impaired Treg inhibition [73]. In prostate cancer, N-cadherin knockout decreased expression of genes related to fatty acid synthesis and the content of free fatty acids in eTreg cells, though the survival time of Treg cells with sufficient free fatty acids was significantly prolonged [74]. In gastric cancer, large amounts of free fatty acids also seem to be a metabolic advantage of Treg cells because these cells consume fatty acids more preferentially and efficiently than CD8+ T cells [75]. Tregs can also increase De novo synthesis of fatty acids and promote maturation and function by up-regulating the activity of steroid regulatory element-binding proteins (SREBPs). However, at the same time, SREBPs also up-regulate expression of PD-1. It was found that down-regulation of SREBP expression by targeted SREBP cleavage activating protein (SCAP) increased apoptosis in Treg cells and could be used in combination with PD-1 blockers to activate an anti-tumour immune response [76].

Compared with CD8+ T cells, the increase in special metabolic pathways, such as fatty acid oxidation, may be one of the main reasons why Treg cells have the advantage of being able to proliferation in hypoxia, low glucose and other harsh environments, which also explains why Tregs induce peripheral immune tolerance and lead to tumour cell immune escape [77, 78]. The energy provided by fatty acid oxidation maintains the survival and differentiation of Treg cells and accelerates fatty acid catabolism through phosphorylation and activation of the AKT/mTor pathway [79]. Studies have found that inhibition of mTor may indeed induce T cells to differentiate into Treg cells and enhance their fatty acid oxidation function [80, 81]. The role of the PPAR signalling pathway was also found in the metabolic adaptation of Tregs. Treg cells highly express CD36 in tumours from cancer patients or mouse models and promote mitochondrial metabolic adaptation by enhancing fatty acid uptake and activating the PPAR-β pathway while reducing metabolic stress caused by an acidified TME [82].

All the above studies suggest that Treg cells in the TME play an important role in tumour immune escape, which requires the coordination of metabolic adaptation. Therefore, regulating the number or function of Treg cells by targeting fatty acid metabolism seems to be an effective means to induce and enhance the antitumour immune response and improve the immunotherapy effect.

### Macrophages and fatty acid metabolism

As an important part of the second line of defence, macrophages can respond quickly to a variety of invading material and physiological challenges. Therefore, macrophages, as innate immune cells, need to maintain stable and flexible functions in the body [83, 84]. In some cancers, macrophages account for the majority of immune cells and show plasticity under different conditions [85]. Macrophages are generally divided into M1 (pro-inflammatory type) and M2 (anti-inflammatory type) according to their function, surface markers and secreted cytokines. Different subsets also have different metabolic characteristics.

Glycolysis and the pentose phosphate pathway (PPP) up-regulation and increased glutamine decomposition and lipogenesis all support the metabolic needs of M1 macrophages [36]. This metabolic regulation assists M1 macrophages in exerting bactericidal and anti-tumour effects. This killing effect on cancer cells is mediated by cytotoxicity or antibody-dependent cytotoxicity (ADCC), but it may cause damage to host tissue [86].

On the other hand, M2 macrophages mainly rely on enhancement of oxidative phosphorylation and β-oxidation of fatty acids to provide ATP, and the biomaterials proline and polyamines depend on metabolism by arginase [87]. Because the TME is rich in fatty acids, M2 TAMs easily increase the degree of fatty acid oxidation [88, 89]. Studies have shown that fatty acid oxidation and lipid accumulation are necessary for the maintenance of the immunosuppressive TAM phenotype [90–92]. Related studies have also described the role of M2 macrophages in tissue remodelling and tumour growth. We believe that M2 macrophages assist tumour proliferation and metastasis and achieve immune escape by antagonizing inhibition of tumour immunity and promotion of tumour angiogenesis [86]. Anti-inflammatory signals such as IL-4, IL-10 and IL-13 are involved in inducing the above processes and bolster fatty acid oxidation and oxidative phosphorylation [93–95]. Fatty acid oxidation induces differentiation of anti-inflammatory macrophages [39]; in the absence of fatty acid transporters, macrophages are more likely to differentiate into pro-inflammatory macrophages [96]. Although researchers are still exploring the significance of fatty acid oxidation leading to M2 polarization of macrophages, it has also been suggested that the use of fatty acids can reduce M1 polarization of macrophages.

Prostaglandin E3 (PGE3) can produce indirect anti-tumour activity by inhibiting the M1 polarization induced by LPS and interferon γ and promoting interleukin-4-mediated M2 polarization. PGE3 selectively promote M2 polarization and inhibit M1 and TAM polarization, thus playing an anti-inflammatory and anti-tumour role in prostate cancer [97]. In head and neck cancer and squamous cell carcinoma, macrophages are polarized by fatty acids in the TME [98]. In prostate cancer, the fatty acid omega-3 inhibits tumour invasion by inhibiting angiogenesis and the T-cell inhibition induced by M2 macrophages, which further highlights the therapeutic potential of host G protein-coupled receptor 120 (GPR120)-dependent omega-3 fatty acids in inhibiting M2 macrophages in prostate cancer [99].

Researchers have confirmed the role of tumour microenvironmental factors in promoting different phenotypes of cells, proved this role of regulating glycolysis-related enzymes in promoting the TAM phenotype, and verified the existence of an inhibitory phenotype by observing high expression of arginase-1 and CXCR1 in the TAM population [100]. It has also been reported that accumulation of unsaturated fatty acids-oleic acid and saturated fatty acids-nonstearic acid and polarization of macrophages lead to an immunosuppressive phenotype. During co-culture of polarized macrophages and CD4+ T cells, the decrease in the proliferation of CD4+ T cells confirmed the effect of oleic acid on the immunosuppressive properties of macrophages, and up-regulation of nitric oxide synthase and arginase-1 was the reason why oleic acid was able to regulate macrophage polarization, playing an immunosuppressive role; arginase-1 simultaneously regulates inhibition of T cells [92, 101]. It has been found that PPAR-γ participates in M2 macrophage polarization [102]; PPARδ inhibits systemic inflammation by improving fatty acid metabolism and insulin sensitivity [103]. High concentrations of linoleic acid and arachidonic acid, agonists of PPARδ, promote M2 polarization of TAMs in the TME [104, 105]. In hepatocellular carcinoma, down-regulation of receptor-interacting protein kinase 3 (RIPK3) promotes fatty acid metabolism by reducing the PPAY cleavage mediated by reactive oxygen species, induces increased M2 polarization of TAMs and accelerates tumour growth [106]. In a mouse breast cancer model, caspase-1 interacts with medium-chain acyl CoA dehydrogenase (MCAD) to cleave PPAR γ at Asp64, inhibit fatty acid oxidation, increase LD accumulation, and promote macrophage differentiation and cancer progression [104]. Fatty acids are often stored in lipid droplets, which can provide PPARδ ligands to up-regulate the target gene of PPAR δ in ovarian cancer to induce TAM polarization [107]. Arachidonic acid is also often stored in lipid droplets of white blood cells. It is the raw material for ab initio synthesis of eicosanoic acid, an inflammatory mediator, and mediates activation, proliferation and inflammation after release [108–110]. As an organelle, lipid droplets store large amounts of fat, play an important role in vesicle transport, protein folding and storage, and act as a signal platform in autophagy [27, 111, 112]. There are not a few studies on LD-mediated fatty acid metabolism, suggesting that targeting lipid droplets as potential cancer therapy is a feasible research direction.

As an efficient drug delivery mode, nano-drug loading has a unique characteristic in affecting fatty acid metabolism in macrophages. We believe that the metabolites of macrophages in different polarization states are important drivers of their signal transduction. It was found that synthesized TLR7/8 agonists and fatty acid oxidation inhibitors are loaded on metabolic supramolecular nano-particles (MSNPs) and delivered to macrophages. In vitro, the phenotype of macrophages changes from M2 to M1 after MSNP treatment, with enhanced phagocytosis. In vivo experiments of 4T1 cell line mice showed that MSNP injection delays tumour growth and enhances anti-tumour immunity [113]. In another group of experiments, siRNA loaded onto a nano-platform with reductive response characteristics was used to silence monoacylglycerol lipase (MGLL) and the key receptor regulating macrophage phenotype (endogenous cannabinoid receptor-2 CBMQ 2), inhibit production of free fatty acids in pancreatic cancer cells and induce TAM reprogramming to M1, increasing secretion of tumour-killing factors (TNF-α, IL-12), both of which exert anti-tumour effects [114]. Although studies have found that direct elimination of macrophages does not have a direct impact on tumour growth [115, 116], specific targeted regulation of TAMs is an effective treatment choice for many diseases. The above experiments reveal the importance of regulating fatty acid metabolism in macrophages or TAMs and provide us with the possibility of targeting fatty acid metabolism in macrophages for cancer immunotherapy.

### Dendritic cells and fatty acid metabolism

Dendritic cells (DC) play an important role in the initiation and persistence of immune response. Dendritic cells are activated by signals transmitted by pathogen-associated molecular patterns (PAMPs) and damage-associated molecular patterns (DAMPs). Dendritic cells present treated antigens to T cells to complete the initiation stage of the immune response. Conventional DCs (cDCs) and plasma cell-like DCs (pDCs) are common phenotypes of DC cells. CDCs mainly promote the anti-tumour T-cell response, whereas pDCs are often related to tumour immunosuppression and immune tolerance [117].

In general, cDCs are activated by TLR agonists and activate the PI3K-AKT and TbK1-IKKε signalling pathways, which promote glycolysis to enhance anabolism and assist in its function [118, 119]. Interestingly, after activation of DCs stimulated by the TLR9 agonist CPGA, glycolysis did not increase but increased FAO and oxidative phosphorylation (OXPHOS), which was induced by DC autocrine type 1 interferon (IFN) and regulated by PPARα [120]. In another study, fatty acid-binding protein 5 (FABP5) was found to regulate TLR signal transduction through the IRF7 and NFκB pathways, and its overexpression promoted Treg production. In contrast, deletion of FABP5 reduced production of pDC cytokines, which decreased Treg production and delayed tumour progression [121]. Fatty acids can also be transmitted by tumour-derived exosomes (TDEs). In many cancer cell lines, fatty acids delivered by TDEs can be induced by PPARα to lead to excessive lipid droplet production, enhance fatty acid oxidation and drive DC immune dysfunction by shifting the metabolic model to mitochondrial oxidative phosphorylation [33]. This is consistent with what we mentioned earlier, indicating that PPAR α may be an effective target for immunotherapy.

The function of DCs can be regulated by fatty acid metabolism reprogramming. High expression of the cDC scavenger receptor MSR1 greatly increases fatty acid uptake, and abnormal accumulation of fatty acids impair the antigen-presenting ability of cDCs; use of acetyl-CoA carboxylase and 5-(tetradecycloxy)-2-furoic acid (TOFA) blocks fatty acid synthesis and restores the T-cell anti-tumour response [122]. Accumulation of oxidized lipids, including fatty acids, in DC cells blocks cross-presentation of antigens by reducing the expression of surface MHCI complexes [123]. In another study, the hyperlipidaemic environment of liver DCs correlated positively with immunogenicity [124]. It is suggested that the effect of lipid content on the function of DCs may be twofold. For example, in an ovarian cancer model, inhibition of FASN reduces fat synthesis and recovers part of the function of DC cells, thus enhancing the anti-tumour immune response [125]. Researchers co-cultured DCs with secreted alpha-fetoprotein (AFP) in hepatocellular carcinoma and found that expression of SREBP-1 and PGC1-α decreased and down-regulated fatty acid synthesis, mitochondrial metabolism and the basal oxygen consumption rate (OCR), which led to DC dysfunction and immunosuppression [126], emphasizing the potential of AFP as a target for immunotherapy of hepatocellular carcinoma.

**Fig. 3:**
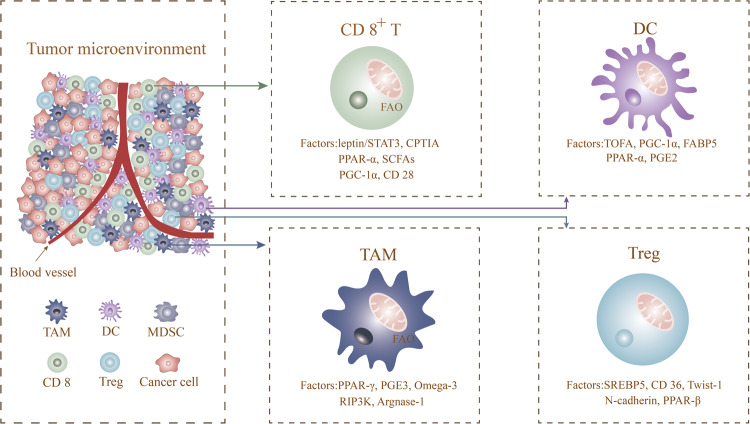
Factors affecting fatty acid metabolism of immune cells in the tumour microenvironment. The tumour microenvironment contains a variety of immune cells, and the factors that affect its fatty acid metabolism are described in this review.

In addition to acting as metabolic substrates, fatty acids have a role as signalling molecules. Prostaglandin E2 (PGE2) is an unsaturated fatty acid produced by tumours that inhibits secretion of the chemokines CCL5 and XCL1 by NK cells, resulting in recruitment of cDCs and a decrease in chemokine receptors in cDCs. Such a decrease in cDC abundance in the TME leads to tumour immune escape (Fig. 3) [127]. The combination of PGE2 and Toll-like receptor agonists (TLRs) can be used as a new standard for DCs with the ability to secrete and migrate cytokines, namely, TLR-P DCs, which successfully induce the response of antigen-specific CD8 T cells to tumour-associated antigens and damage cross-presentation of protein antigens in human DCs and CD8 T cells in a dose-dependent manner [128]. Therefore, blocking the PGE2 signalling pathway can reverse tumour immune escape, which may be a promising target for tumour immunotherapy. The above findings show that enhancement of fatty acid synthesis and accumulation of fatty acids in the TME hinder the function of tumour-related DCs, resulting in a decrease in the ability of DCs to activate T cells to induce an anti-tumour immune response. Therefore, fatty acid metabolism targeting cancer DCs may have a positive therapeutic effect on improving the immunosuppressive tumour microenvironment.

## Fatty acid metabolism and tumour immunotherapy

**Fig. 4:**
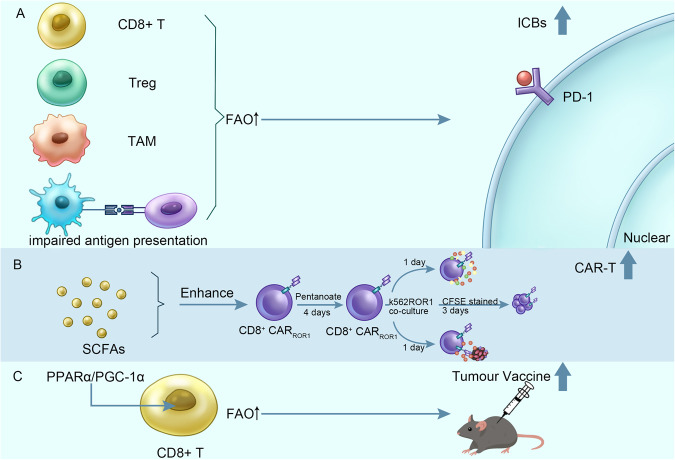
Application of fatty acid metabolism in tumor immunotherapy. **A** Immune cells enhance fatty acid metabolism through corresponding targets, thus increasing the efficacy of ICBs. **B** Some short-chain fatty acids enhanced the efficacy of CD8^+^T cells treated with CAR-T. **C** Promote the combination of immune cell fatty acid metabolism and tumor vaccine.

In recent years, researchers have regarded changes in fatty acid metabolism and related signals as markers of cancer and have revealed their importance for cancer progression through experimentation. Particularly in the TME, various fatty acids may play an important role in activating the anti-tumour immune response by regulating the function and differentiation of immune cells. Considering the role of fatty acid metabolism in the pathogenesis and progression of tumours, changes in fatty acid metabolism in immune cells are often regulated by various signals in tumour cells and further induce immune escape. Therefore, it is very important to explore how tumours affect the fatty acid metabolism of immune cells in the TME and to study the reprogramming of fatty acid metabolism as a target. In this section, we review studies on the relationship between fatty acid metabolism and tumour immunotherapy and explore the possibility of further development of immunotherapy (Fig. 4).

Immune checkpoint blockade (ICB) therapy is often referred to as an innovation in the field of cancer treatment and shows significant efficacy in many types of tumour treatment, but only approximately 1/4 of cancer patients can benefit from ICB because of immunosuppression and immune tolerance [129–131]. Encouragingly, one study found that fatty acid metabolism of targeted immune cells improves the efficacy of ICB. It was found that fenofibrate, a PPAR-α agonist, enhances the anti-tumour function of CD8+ TILs in mouse colon cancer and synergizes with a PD-1 blocker [132]. Similarly, PPAR-γ co-activator 1α (pGC-1α) has a synergistic effect with PD-1 blockers and may be achieved by up-regulating CPT1, which is necessary for the oxidation of CTL fatty acids, and forming a complex with Bcl2 to prevent CTL apoptosis [133]. In a recent study of a mouse Lewis lung cancer cell model, the pGC-1 α activator bezafibrate maintained the cell survival and function of CTLs by increasing expression of fatty acid oxidation-related genes (PGC-1α, CPT1A) in CTLs, and synergistic therapy with an anti-PD-L1 antibody enhanced its tumour-killing ability [134]. These experimental results show that pGC-1α agonists combined with PD-1/PD-L1 antibodies enhance the tumour-killing ability of CD8+ TILs and support the great potential of PPAR as a fatty acid metabolism target, as mentioned above.

Using metabolic drugs to modulate the immunosuppressive TME has been shown to be an effective therapeutic approach. Studies have shown that M2 TAMs and Treg cells enhance the immunosuppression of the tumour microenvironment and promote immune escape. Experiments have shown that Treg cells enhance the immunosuppressive TME by inhibiting the secretion of IFN-γ by CD8+ T cells, which is the basis of the TAM-M2 polarization induced by SREBP1 mediated by fatty acid synthesis [135]. In a tumour-bearing mouse model, an SREBP1 inhibitor led to a large lack of free fatty acids and resulted in an increase in CD8+ T cells and a decrease in M2 TAMs under combined treatment with anti-PD-1 therapy, enhanced antitumour immunity and inhibited tumour growth [135]. At the same time, Treg cells in the TME express the SREBP gene to mediate the antitumour immune response. FASN-mediated fatty acid synthesis and SREBP cleavage-activating protein (SCAP) cooperate to regulate PD-1 gene expression and maintain Treg cell inhibition [76]. It has been suggested that the SCAP/SREBP signalling pathway has a synergistic inhibitory effect with anti-PD-1 therapy in M2-TAM and Treg cells and enhances anti-tumour immunity.

In another study, detecting the concentration of ultra-long-chain fatty acids (VLCFAs) in serum was able to assess the therapeutic response of urinary cancer patients treated with immune checkpoint inhibitors (nivolumab or atezolizumab), which may be related to the fatty acid catabolism of immune cells [136]. There is a great deal of evidence that the intestinal microflora is closely related to response to immunotherapy [137–140]. Short-chain fatty acids (SCFAs), such as acetate, propionic acid and butyrate, are the main end products of intestinal microflora. High levels of single-chain fatty acids are significantly associated with better outcomes in patients receiving anti-PD-1 therapy [141, 142]. The ability of SCFAs to regulate immune function may explain the ability to predict treatment outcome, but whether it is also involved in a change in treatment outcome needs to be revealed by more studies.

Tumour growth was significantly inhibited after a patient’s white blood cells and tumour cells were infused back into the body. This treatment, which was first carried out in 1966, was later called adoptive cell therapy (ACT) [143]. Recently, many studies have suggested that fatty acid metabolism can regulate the memory stroke of T cells and play a vital role in maintaining the long-term efficacy of transplanted cells [45, 144, 145]. In vivo, TILs cultured with AKT inhibitors enhance memory characteristics and show enhanced persistence after adoptive transfer [146]. Mechanistically, accumulation of long-chain and polyunsaturated fatty acids leads to enhanced fatty acid oxidation in AKI-treated CD8+ T cells, and up-regulation of FAO enhances mitochondrial respiration. The use of AKI enhances anti-tumour immunity by promoting\differentiation of T cells into memory subsets and improving the efficiency of adoptive TIL [146]. On the other hand, activation of PPAR δ and PPAR α promotes FAO in activated CD8+ T cells, and enhanced production of IFN γ maintains the persistence and good function of T cells in the ACT model [60]. This is consistent with the report that CPT1 inhibitors combined with adoptive cell therapy show more significant anti-tumour function [147].

Chimeric antigen receptor T-cell (CAR-T) therapy is a kind of adoptive T-cell therapy that has been proven to have excellent therapeutic potential in haematological cancer [148, 149], but its application in solid tumours still faces great challenges [150]. Recent studies have found that the short-chain fatty acids (SCFAs) valerate and butyrate inhibit histone deacetylase (HDACs), increase production of effector factors (CD25, IFN-γ and TNF-α) and enhance the antitumor activity of receptor tyrosine kinase-like orphan receptor 1 (ROR1) targeting CAR T cells and CTLs in mouse melanoma and pancreatic cancer adoptive metastasis models [55], which has a positive effect on the clinical application of adoptive T-cell therapy.

The first batch of therapeutic cancer vaccines has been approved, and although they have performed well in enough preclinical trials, their clinical application in cancer patients has not yielded gratifying results [151]. The therapeutic cancer vaccine did not improve the overall and disease-free survival of patients with MAGE-A3-positive non-small cell lung cancer (MAGRIT) during phase 3 treatment [152]. Although we still do not fully understand the causes of tumour immune escape and immunosuppressive TME formation, considering the role of fatty acid metabolism in affecting immune capacity, we may be able to focus on combining therapeutic cancer vaccines with drugs targeting fatty acid metabolism of immune cells in the TME to enhance immune activity. Studies have shown that overexpression of PGC-1α increases differentiation of CD8 central memory T cells and that increased fatty acid oxidation in T cells enhances SRC, mediates stronger peptide vaccine memory ability and promotes anti-tumour immunity [153]. In a mouse melanoma model, tumour progression was delayed by the combination of a PPARα agonist and a cancer vaccine. Among them, the increased demand for fatty acids by tumour and stromal cells increases the chance of glucose uptake by T cells, which in turn enhances anti-tumour immunity [154]. According to the above experiments, the combination of therapeutic cancer vaccines and immune cell FA metabolic drugs appears to be a feasible strategy for cancer treatment and should be considered together with other immunotherapy methods; further research should be carried out (Table 2).

**Table 2:** Cancer immunotherapy associated with fatty acid metabolism.

Model	Drug/Treatment	Mode of combined treatment	Metabolic changes	Effect	Reference
Colon cancer	PPAR-gamma coactivator 1α (PGC-1α)	PD-1 blockade	Activates fatty acid oxidation and OXPHOS	Enhances tumour-growth suppression	[132]
Lewis lung carcinoma	PPAR-gamma coactivator 1α (PGC-1α)	Anti-PD-L1	Induces fatty acid oxidation in tumour-infiltrating CTLs	Promotes infiltration of CD8^+^T cells into tumour tissue and enhances the antitumor effect.	[134]
Breast cancer, head and neck squamous cell carcinoma	Sterol regulatory element-binding protein (SREBP)	PD-1 blockade	Promotes de novo synthesis of lipids including fatty acids and cholesterol	Inhibits tumour growth and boosts anti-PD-1 immunotherapy	[76]
Melanoma	AKT inhibition	Adoptive cell therapy (ACT)	Enhances fatty acid oxidation and persistence of TILs	Augments anti-tumour immunity of CD8 T cells	[146]
Pancreatic cancer	PPAR agonist GW501516	Adoptive cell therapy (ACT)	Increases expression of CPT1A, consistent with enhanced FAO.	Maintains the persistence and good function of T cells in the ACT model	[60]
Melanoma	SCFAs (pentanoate and butyrate)	Chimeric antigen receptor T-cell (CAR-T) therapy	Results in elevated production of effector molecules such as CD25, IFN-γ and TNF-α	Enhances the anti-tumour activity of human CAR-T cells.	[55]
Melanoma	PPAR-gamma coactivator 1α(PGC-1α)	Peptide vaccine	Activate fatty acid oxidation and OXPHOS	Enhances recall response to peptide vaccination	[153]
Melanoma	PPARα agonist Fenofibrate	Cancer vaccine	Increases fatty acid metabolism in tumour cells	Increases the efficacy of cancer vaccines and slow tumour progression	[154]

## Conclusion

There may be metabolic competition for limited nutrients between tumour cells and immune cells, which is an important feature of the TME and has a great impact on immunotherapy and the clinical response of patients. Through metabolic adaptation to a variety of adverse and dynamic environments, tumour cells gradually show the ability to absorb a variety of nutrients to meet their growth needs. There has been increasing evidence that cancer provides energy and even information to tumour cells in harsh environments through fatty acid metabolism.

In this review, we summarize the latest research on fatty acid metabolism in immune cells in the TME and the application and prospects of fatty acid metabolism in tumour immunotherapy. Overall, the synthesis, decomposition, uptake and transmission of fatty acids participate in all levels of immune cell functions (including killing, activation and differentiation) through a large number of substrates from their own or other sources through metabolic pathways rather than simply providing energy. The metabolic characteristics of the tumour microenvironment characterized by acidification, hypoxia and energy depletion reveal the application of fatty acids, and performance in immune cells is even more impressive. For example, the use of short-chain fatty acids (SCFAs) can enhance the anti-tumour activity of CAR T cells and CTL cells by increasing the production of effector factors, which has unique therapeutic potential.

Considering the improvement of immunotherapy strategies, inhibition of a single pathway or a single enzyme does not reflect the outstanding potential of targeted fatty acid metabolism in cancer immunotherapy. Therefore, the combination of fatty acids and their synthesis, functional pathway and even the whole tumour immune microenvironment as the target may provide a direction for the implementation of the new combined therapy strategy. Hence, tumour immunotherapy strategies, including immune checkpoint blocking, adoptive cell therapy and cancer vaccines, can target the dependence of tumour cells on fatty acids in the form of drug targeting. This approach was combined with the traditional model to exert a synergistic anti-tumour effect. However, due to the different types of cancer and the uniqueness of the TME, it is still a problem to find accurate targets and reduce possible side effects. Therefore, it is of great significance to explore the target of fatty acid metabolism of immune cells in the TME to enhance the ability of anti-tumour immunotherapy, and in the future, more researchers will devote efforts to understanding the function and interaction of immune cells and fatty acid metabolism in the TME to find a new way to improve the efficiency of tumour immunotherapy.
